# Cutting time & height improve carbon and energy use efficiency of the forage–food dual-purpose ratoon rice cropping

**DOI:** 10.1016/j.heliyon.2023.e14042

**Published:** 2023-02-24

**Authors:** Yuanwei Chen, Huabin Zheng, Weiqin Wang, Qiyuan Tang

**Affiliations:** a College of Agronomy Hunan Agricultural University, Changsha 410128, PR China; b Collaborative Innovation Center for Grain and Oil Crop in Southern Paddy Field, Changsha 410128, PR China

**Keywords:** Ratoon rice, Forage yield, Grain yield, Carbon footprints

## Abstract

Forage–food dual-purpose ratoon rice cropping (FFRR) is used to balance forage demands with ratoon rice grain yields, that is whole plant (stem and sheath, panicles) cuttings in the main season are used as forage, and rice in the regeneration season is used as food. In this study, the local ratoon rice production system as the control, we were carried out the field experiment of cultivation practices (cutting time and cutting height), and investigated the system productivity, economic benefits, carbon footprints and energy use efficiency. The energy use efficiency, energy productivity and energy profitability increased with cutting time delay, and cutting height decreased. Significant differences of these index were observed among the treatments for cutting time and cutting height (p < 0.05). Carbon efficiency and carbon sustainability index was increase with cutting time delay, and there was significant difference among the treatment of cutting time in 2018 (p < 0.05), the minimum carbon footprint of FFRR was 78.6 kgCO_2_ t^−1^ averagely at the cutting time of 30 days after the flowering stage. In 2018, the maximum net income of FFRR was 30,577 CNY hm^−2^ at a cutting time of 30 days after the flowering stage while the stubble height was 10 cm, and dependent on the forage yield of the main crop; in 2019, the maximum net income of FFRR was 27,326 CNY hm^−2^ at a cutting time of 10 days after the flowering stage while the stubble height was 10 cm, and dependent on the grain yield of the ratoon crop. Therefore, the optimal cultivation practice of the FFRR (cutting at 30 days after the flowering stage and with a stubble height of 10 cm) showed higher carbon and energy use efficiency, economic benefits of the FFRR were fluctuated with the price of forage of the main crop and rice grain of the ratoon crop.

## Highlights

• To balance forage demands with ratoon rice grain yields by forage–food dual-purpose ratoon rice cropping (FFRR).
• Cutting time delay and cutting height decreased showed higher carbon and energy use efficiency.
• FFRR is an effective way to ensure both forage security and unprocessed grain security in the impoverished regions.

## Graphical abstract

**Figure:**
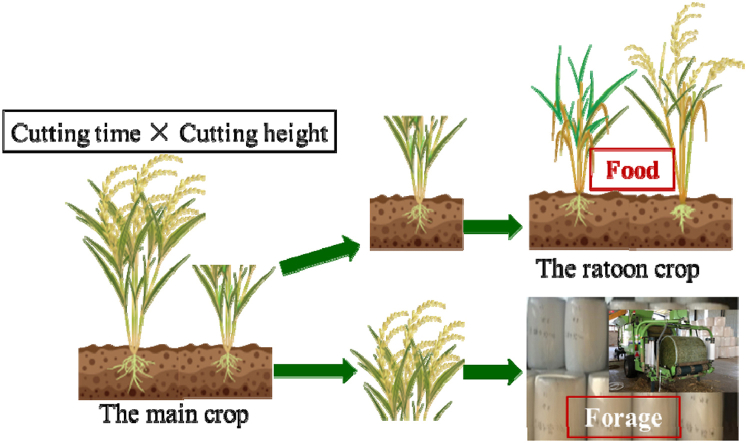

## 1 Introduction

It needs to adjust its agricultural production system to balance cereal production and livestock farming [1]. According to statistics, the food (e.g., rice and wheat grain) consumption per person declined from 138.9 kg in 2013 to 117.9 kg in 2019. In particular, rice was food for more than 60% of the population in China, and the annual consumption of red meat (pork, beef, and mutton) per person grew from 19.8 kg in 2013 to 26.9 kg in 2019 (China Statistical Yearbook). China's red meat consumption will continue to grow into the future and stimulate the development of livestock farming. Large amounts of forage are needed to feed domestic animals; consequently, rice straw has been widely perceived as an important source of forage. The average planting area of the rice (*Oryza sativa* L.) production system is 6 million hm^−2^, and this system needs to meet the developing food structure needs of Chinese diets.

Ratoon rice is a second rice crop produced from the stubble left behind after the main crop has been harvested [2], and there is more than 1 million hm^2^ of ratoon rice cropping in southern China [3]. Rice ratooning is associated with a significantly larger net energy and cost–benefit ratio and substantially lower global warming potential than are middle- and double-season rice cultivation techniques [4]. Reference [5] reported that developing ratoon rice as forage could increase forage yields without placing extra demand on fields in subtropical and temperate rice areas, but it does not balance cereal production with forage production for livestock farming. Based on the need for forage and improvements in ratoon rice grain yields, consequently, we propose a new ratooning rice cropping model, a forage–food dual-purpose ratoon rice cropping (FFRR) model, to balance forage demands with high ratoon rice grain yields.

Cutting time and stubble height are two important factors affecting the rice yield and quality of the ratoon crop, and affecting forage (straw) yield of the main crop. For example, Ref. [6] reported that significant differences in ratoon rice grain yield were observed with cutting time and cutting height of the main crop, and that the highest grain yield was found at a stubble height of 40 cm. Reference [7] reported that the rice quality (such as the amount of head rice and protein content) of main crops were significantly lower than those of ratoon crops. However, Previous studies was concentrated on grain yield of the ratoon crop by different cutting time and stubble height, and the report about the effect of forage yield on different cutting time and stubble height was few. Therefore, the scientific hypotheses of the study were that how balancing rice straw as forage and rice grain as food according to the cutting time and stubble height of the main crop would be beneficial for the development and improvement of ratoon rice production.

In Hunan Province, the cutting time and stubble height in the ratoon rice production system are about 30 days after the flowering stage and 30 cm, the annual grain yield is about 12–15 t hm^−2^, and the ratio of the grain yield between the main crop and ratoon crop is 7:3 or 8:2. However, poor rice quality (high temperatures during the filling stage) of the main crop and a lower grain yield of the ratoon crop have affected rice producers’ income, although ratoon rice attains a higher annual grain yield, net energy yield, and net economic return than middle-season rice. In this study, based on a new ratooning rice cropping model (FFRR), compared with the traditional ratoon rice production system, with regard to total annual yields, energy, profits, and environmental footprint, the goal of this study was to investigate the effects of cultivation practices (cutting time and cutting height) on the forage yield of the main crop (the whole plant exclude stubble) and the rice grain yield of the ratoon crop to provide direction for agricultural practice.

## 2 Materials and methods

### 2.1 Ratoon rice planting

FFRR was established in Yiyang City, Hunan Province, (29°08′N, 112°26′E) in 2018–2019. Its basic climate condition was characterized by annual average sunshine time of 1643.3 h, annual average temperature of 16.9 °C, the coldest month (January) has an average temperature of 4.3 °C and the hottest month (July) an average temperature of 29.1 °C, frost-free period of 264 days, accumulated temperature (≥10 °C) of 5240 °C, and an average rainfall of 1240.8 mm, concentrating in the period from May to September. The rice cultivars were Xiangliangyou 900 (XLY900) that was widely planting in the Hunan Province, and the planting area was 1 hm^2^. The detail of these rice cultivar characteristics was posted on the China rice data center (www.ricedata.cn).

Sowing was done on March 29th, and the seed quantity was 22.5 kg hm^−2^. At a seedling age of 30 days, machine transplanting was performed (on April 29th). The nitrogen (N) application rate was 370 kgN hm^−2^ (220 kgN hm^−2^ for the main crop and 150 kgN hm^−2^ for the ratoon crop), in accordance with the local high-yield and high-quality ratooning rice cultivation system. The P_2_O_5_ and K_2_O application rates were 90 and 225 kg hm^−2^, respectively.

**Fig. 1:**
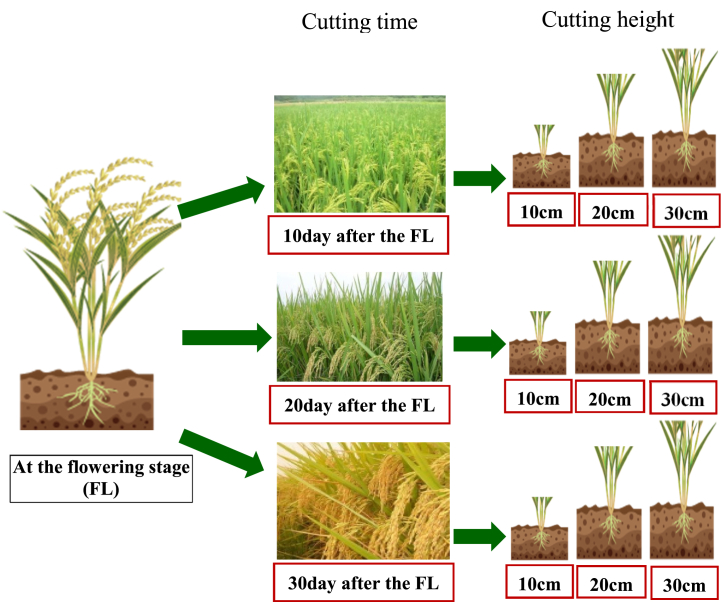
Schematic diagram of the forage–food dual-purpose ratoon rice cropping in the experiment.

Begin with the flowering stage of the main crop, three cutting times (10, 20, and 30 days after the flowering stage) were tested. Two cutting heights (stubble of 10 and 30 cm) were used per cutting time, respectively, these variants were applied in three replications with an area of 500 m^2^ per the treatment (Fig. 1). Begin with 10 days after the flowering stage in the main crops, rice plant exclude the rice stubbles, as forage, was harvested using a combine harvester. The local ratoon rice production system as the control, at the mature stage of the main crop, the rice grain was harvested using a combine harvester, and the cutting time and heights were 30 days after the flowering stage and 30 cm stubble. At the mature stage of the ratoon crop, the rice grain was also harvested using a combine harvester.

Pesticides, including those used to prevent rice planthopper, and damage from rice borers. Herbicides was used for controlling barnyard grass, and fungicide, including those used for seed disinfection and sheath blight controlling. Other cultivation measures followed the local high-yield and high-quality ratooning rice cultivation system.

### 2.2 Data records

Records of every input and output in the FFRR were kept as raw data for assessing the carbon budget, energy, and economics. The input and output data, including labor (adult men), fertilizers, diesel fuel, electric motors, plastics (general), pesticides, fungicides, herbicides, irrigation water, rice seed, rice straw yield, and rice grain yield were calculated and recorded. The inputs of the FFRR were the same in 2018 and 2019. The output data in 2018–2019 were used as average values. The carbon transformity [8,9] and energy transformity [10] and the prices of materials during the years were as listed in Table 1. Experimental research and field studies on cultivated plants, including the collection of plant material, must comply with relevant institutional, national, and international guidelines and legislation.

**Table 1:** The carbon and energy transformity, the price of the materials in the forage–food dual-purpose ratoon rice cropping.

Items	Energy equivalences^a^	Carbon equivalences^b^	Price
Renewable source (R)			
Irrigation	4.18 MJ t^−1^		195.00 CNY hm^−2^
Renewable organic subsidiary sources (R1)			
Human			
Adult man	1.96 MJ h^−1^	0.860 kgCO_2_ h^−1#^	150.00 CNY/(8hr.person)
Rice seed	16.74 MJ kg^−1^	0.577 kgCO_2_ kg^−1^	50.00 CNY kg^−1^
Nonrenewable industrial subsidiary sources (F)			
N	61.39 MJ kg^−1^	1.530 kgCO_2_ kg^−1^	1.70 CNY/kg
P_2_O_5_	14.31 MJ kg^−1^	1.630 kgCO_2_ kg^−1^	0.60 CNY/kg
K_2_O	9.40 MJ kg^−1^	0.655 kgCO_2_ kg^−1^	2.50 CNY/kg
Diesel	47.79 MJ L^−1^	0.886 kgCO_2_ L^−1^	6.40 CNY L^−1^
Plastics-general	90.00 MJ kg^−1^	22.700 kgCO_2_ kg^−1^	10.00 CNY kg^−1^
Insecticide	303.25 MJ kg^−1^	16.600 kgCO_2_ kg^−1^	235.34 CNY kg^−1^
Herbicides	310.25 MJ kg^−1^	10.200 kgCO_2_ kg^−1^	300.00 CNY kg^−1^
Fungicides	185.00 MJ kg^−1^	10.600 kgCO_2_ kg^−1^	27.00 CNY kg^−1^
			
Rice grain	15.19 MJ kg^−1^	0.450kgC kg^−1^	0.70 CNY kg^−1^
Rice straw	13.40 MJ kg^−1^	0.450kgC kg^−1^	0.70 CNY kg^−1^

a , Zheng et al., 2014; Lal et al., 2019.

b ISO14040, Krozer & Vis, 1997.

### 2.3 Energy budgeting and related indices

The indices used for the energy assessments included input energy (Ei), output energy (Eo), renewable organic subsidiary sources (R1), nonrenewable industrial subsidiary sources (F), net energy (NE = Ei − Eo), energy use efficiency (EUE = Eo/Ei), energy productivity (EP = system productivity/Ei), and energy profitability (PE = NE/Ei).

### 2.4 Carbon footprint and related indices

The carbon transformity (Table 1) was used to calculate the carbon footprints (CFs) of the various inputs. Rice straw, grain, and root were used to calculate the carbon quantity of the various outputs. The harvest index of the ratoon crops was 0.45, the ratio of root to aboveground portion was 0.10, and the transformation coefficient of biomass and its carbon content was 0.42 (grain) and 0.38 [11]. The summations of the inputs and outputs were represented as the total carbon input and output, respectively. The indices included crop primary productivity (CPP = the sum of all outputs), carbon footprint (CFy = CFs/system productivity), carbon efficiency (carbon output/carbon input), and carbon sustainability index (CSI = [carbon output/carbon input] − 1).

## 3 Economic benefits and related indices

Refer to the method of [8] the economic indices used in the study were gross income (GI), net income (NI), output/input ratio, cost–benefit ratio (NI/input), and labor productivity (NI/quantity of labor input).

### 3.1 Data analysis

Means were calculated using Microsoft Excel 2007 software. We performed a factorial analysis of variance and a least squares difference to test for statistically significant differences between days after the flowering stage and stubble height using Statistix 8.0.

## 4 Results

### 4.1 Inputs of the FFRR

Three metrics, energy input, equivalent carbon emissions for agricultural inputs, and inputs based on money, were used to describe the inputs of the FFRR (Table 2). The total energy equivalent of the FFRR was 33,993.0 MJ hm^−2^. The energy equivalent of the nonrenewable industrial subsidiary source (F) was 343,316.1 MJ hm^−2^, accounting for 88.9% of the FFRR, and that of the renewable source (R) and renewable organic subsidiary source (R1) was 3825.5 MJ hm^−2^. The energy equivalent of N was 22,714.3 MJ hm^−2^, which accounted for 66.2% of the FFRR.

**Table 2:** Estimates of energy inputs, equivalent carbon emissions for agricultural inputs used and input based on money in the forage–food dual-purpose ratoon rice cropping.

Items	Unit	Quantity (hm^2^)	Energy equivalent (MJ hm^−2^)	Equivalent carbon emissions (kgCO_2_ eq hm^−2^)	Input based on money (CNY hm^−2^)^b^
Renewable source (R)					
Irrigation water	t	600.00	2508.0 (7.31)		195.0
**Renewable organic subsidiary sources (R1)**					
Labor					
Adult man^b^	hr	480.00	940.8 (2.74)	412.8 (27.75)	9000.0
Rice seed	kg	22.50	376.7 (1.109)	13.0 (0.87)	1125.0
**Nonrenewable industrial subsidiary sources (F)**					
N	kg	370.00	22714.3 (66.19)	566.1 (38.05)	1349.8
P_2_O_5_	kg	90.00	1287.9 (3.75)	146.7 (9.86)	442.6
K_2_O	kg	225.00	2115.0 (6.12)	147.4 (9.91)	937.5
Diesel	kg	70.50	3369.2 (9.82)	62.5 (4.20)	451.2
Plastics-general	kg	5.00	450.0 (1.31)	113.5 (7.63)	50.0
Insecticide	kg a.i.^c^	0.58	175.9 (0.52)	9.6 (0.65)	235.3
Herbicides	kg a.i.	1.13	350.6 (1.02)	11.5 (0.77)	300.0
Fungicides	kg a.i.	0.15	27.8 (0.08)	1.6 (0.11)	27.0
**Total**			**34316.1(100.0)**	**1484.7(100.0)**	**14113.4**

The numerical value of the bracket was percentage. ^b^1CNY = 0.1571U.S.$. ^c^a.i. active ingrindient.

The equivalent carbon emission for the agricultural inputs of the FFRR was 1424.0 kgCO_2_ eq. hm^−2^, and that of the nonrenewable industrial subsidiary source (F) was 1058.9 kgCO_2_ eq. hm^−2^, accounting for 71.3% of the FFRR. The carbon footprint of fertilizer (including N, P_2_O_5_, and K_2_O) was 806.2 kgCO_2_ eq. hm^−2^, accounting for 60.3% of the total. Inputs based on money of the FFRR were 14,113.4 CNY hm^−2^.

### 4.2 Outputs of the FFRR

The straw yield of the main crop increased with cutting time delay and decreased with cutting height increase. A significant difference was observed among different cutting times (T, *p* < 0.01) and cutting heights (H, *p* < 0.01), but the interaction produced no significant difference between T and H. The maximum straw yields of the main crop were 15,438 kg hm^−2^ in 2018 and 18,192 kg hm^−2^ in 2019 at 30 days after the flowering stage while the stubble height was 10 cm. However, the variation in grain yield of the ratoon crop was opposite the straw yield result of the main crop. The grain yield of the ratoon crop decreased with cutting time delay and increased with cutting height increase. The maximum grain yields of the ratoon crop were 6633 kg hm^−2^ in 2018 and 7199 kg hm^−2^ in 2019 at 10 days after the flowering stage while the stubble height was 30 cm, and significantly higher (*p* < 0.01) than that of the local ratoon rice production model.

The average content of crude protein in the rice straw was 9.35% (range from 8.05% to 10.66%), and the average total crude protein was 1630 kg hm^−2^ (from 1348 to 1911 kg hm^−2^), while that of the local ratoon rice production model was 1229 kg hm^−2^ averagely. The variance analysis revealed that there were no significant differences in crude protein content among the treatments of T, H, and T × H (*p* > 0.05, Table 3); and significant differences in total crude protein among the treatments of T (*p* < 0.05).

**Table 3:** Variation of straw yield of main rice and grain yield of ratoon rice among different cutting time and cutting height in the forage–food dual-purpose ratoon rice cropping.

Year	Cutting time (days after FL)^#^	Cutting height (cm)^#^	Forage/grain of main crop		Grain of ratoon crops		Total crude protein (kg hm^−2^)
			Straw yield (kg hm^−2^)	Protein content (%)	Grain yield (kg hm^−2^)	Protein content (%)	
2018	10	10	9602a	8.05a	5720b	9.56a	1317a
		30	7610b	9.85a	6633a	9.52a	1379a
	**Average**		**8606C**	**8.95A**	**6176A**	**9.54A**	**1348C**
	20	10	12488a	8.33a	4755b	9.25a	1478a
		30	9970b	10.16a	6052a	9.21a	1570a
	**Average**		**11229B**	**9.24A**	**5404C**	**9.23B**	**1524B**
	30	10	15438a	9.21a	4426b	9.21a	1829a
		30	11837b	9.36a	5618a	9.24a	1634a
	**Average**		**13637A**	**9.29A**	**5022C**	**9.23B**	**1732A**
	Local ratoon rice production model^&^		**7694D**	**9.43A**	**5618D**	**9.24B**	**1244C**
ANOVA	Cutting time (T)		633**	ns	138**	0.05**	153**
	Cutting height (H)		610**	ns	107**	ns	ns
	T*H		ns	ns	185*	ns	ns
2019	10	10	10321a	8.57a	6350b	9.57a	1492a
		30	7166b	9.66a	7199a	9.46a	1377a
	**Average**		**8744C**	**9.11A**	**6774A**	**9.52A**	**1434C**
	20	10	14511a	9.18a	4491b	9.45a	1757a
		30	11331b	10.66a	4949a	9.34a	1670a
	**Average**		**12921B**	**9.92A**	**4720B**	**9.39A**	**1713B**
	30	10	18192a	10.13a	2625b	9.47a	2097a
		30	14084b	9.61a	3840a	9.55a	1725a
	**Average**		**16138A**	**9.87A**	**3233D**	**9.51A**	**1911A**
	Local ratoon rice production model		**8848C**	**9.56A**	**3840C**	**9.55A**	**1212D**
ANOVA	Cutting time (T)		807**	ns	154**	ns	168**
	Cutting height (H)		744**	ns	125**	ns	163*
	T*H		ns	ns	217**	ns	ns

^#^Different treatment of the main season of ratooning rice.

^&^Cutting time and heights of the local ratoon rice production model was 30 cm stubble height at the mature stage of the main crop.

Different small letter between means were assessed with the least significant difference (LSD0.05) at p ≤ 0.05.

* means significant at the 0.05 level, ** means significant at the 0.01 level, and ns means the correlation is not significant. The same below.

### 4.3 Energy budget of the FFRR

The maximum total output energy of the FFRR was 274,099 MJ hm^−2^ in 2018 and 283,654 MJ hm^−2^ in 2019 with the treatment of 30 days after the flowering stage while the stubble height was 10 cm, and the variation in net energy was the same as the variation in the total output energy (Table 4). The variance analysis showed that there were significant differences in total output energy and net energy among the cutting time and cutting height treatments (*p* < 0.05, Table 4). Compared with the local ratoon rice production model, FFRR was shown high total output energy and net energy.

**Table 4:** Energy output and its index among different cutting time and cutting height in the forage–food dual-purpose ratoon rice cropping.

Year	Cutting time (days after FL)	Cutting height (cm)	Output energy (MJ hm^−2^)			Net energy (MJ hm^−2^)	EUE	EP	PE
			Straw/grain yield of main rice	Grain yield of ratoon rice	Total				
2018	10	10	128663a	86881b	215543a	181227a	6.28a	0.45a	5.28a
		30	101972b	100748a	202720a	168404a	5.91a	0.42a	4.91a
	**Average**		**115317C**	**93815A**	**209132C**	**174816C**	**6.09C**	**0.43C**	**5.09C**
	20	10	167345a	72235b	239580a	205264a	6.98a	0.50a	5.98a
		30	133601b	91928a	225529b	191213b	6.57b	0.47b	5.57b
	**Average**		**150473B**	**82082C**	**232554B**	**198238B**	**6.78B**	**0.49B**	**5.78B**
	30	10	206866a	67233b	274099a	239783a	7.99a	0.58a	6.99a
		30	158614b	85335a	243949b	209633b	7.11b	0.51b	6.11b
	**Average**		**182740A**	**76284D**	**259024A**	**224708A**	**7.55A**	**0.55A**	**6.55A**
	Local ratoon rice production model^&^		**116872C**	**85335B**	**202206C**	**167890C**	**5.89C**	**0.39D**	**4.89C**
ANOVA	Cutting time (T)		8455**	2089**	8912**	8912**	0.26**	0.02**	0.26**
	Cutting height (H)		8180**	1620**	8566**	8566**	0.25**	0.02**	0.25**
	T*H		ns	2806*	ns	ns	ns	ns	ns
2019	10	10	138306a	96452b	234759a	200443a	6.84a	0.49a	5.84a
		30	96022b	109355a	205376b	171060b	5.99b	0.42b	4.99b
	**Average**		**117164D**	**102904A**	**220068C**	**185752C**	**6.42C**	**0.45C**	**5.42C**
	20	10	194452a	68218b	262670a	228354a	7.66a	0.55a	6.66a
		30	151838b	75168a	227006b	192690b	6.61b	0.48b	5.61b
	**Average**		**173145B**	**71693B**	**244838B**	**210522B**	**7.14B**	**0.52B**	**6.14B**
	30	10	243776a	39878b	283654a	249338a	8.27a	0.61a	7.27a
		30	188721b	58324a	247045b	212729b	7.20b	0.520b	6.20b
	**Average**		**216249A**	**49101D**	**265350A**	**231034A**	**7.73A**	**0.56A**	**6.73A**
	Local ratoon rice production model		**134401C**	**58324C**	**192725D**	**158409D**	**5.62D**	**0.37D**	**4.62D**
ANOVA	Cutting time (T)		10,951**	2341**	10,166**	10,166**	0.30**	0.02**	0.30**
	Cutting height (H)		9974**	1902**	9530**	9530**	0.28**	0.02**	0.28**
	T*H		ns	3295**	ns	ns	ns	ns	ns

EUE, Energy use efficiency; EP, Energy productivity; PE, Energy profitability; NE, Net energy.

Different small letter between means were assessed with the least significant difference (LSD0.05) at p ≤ 0.05.

^&^Cutting time and heights of the local ratoon rice production model was 30 cm stubble height at the mature stage of the main crop.

*means significant at the 0.05 level, ** means significant at the 0.01 level, and ns means the correlation is not significant.

The energy use efficiency, energy productivity and energy profitability increased with cutting time delay and cutting height decreased (Table 4). Significant differences of these index were observed among the treatments for cutting time and cutting height (p < 0.05). The interaction between T and H also revealed no significant differences. Compared with the local ratoon rice production model, FFRR was shown high energy use efficiency, energy productivity and energy profitability.

### 4.4 Carbon budget of the FFRR

The maximum CPP of the FFRR were 11,065 kgC hm^−2^ in 2018 and 10,629 kgC hm^−2^ in 2019 in the treatment of 30 days after the flowering stage while the stubble height was 10 cm, and there were no significant differences among the treatments of T and H (Table 5). There was a significant difference in carbon footprint among the treatments of days after flowering stage. The minimum carbon footprint of the FFRR was 74.8 kgCO_2_ t^−1^ in 2018 and 71.4 kgCO_2_ t^−1^ in 2019 in the treatment of 10 days after the flowering stage while the stubble height was 30 cm, while there was significant difference (p < 0.05) between the treatment of 30 days after the flowering stage while the stubble height was 10 cm and the local ratoon rice production model (Table 5).

**Table 5:** Carbon footprint and its index among different cutting time and cutting height in the forage–food dual-purpose ratoon rice cropping.

Year	Cutting time (days after FL)	Cutting height (cm)	CPP (kg C/hm^2^)	CFs (kgCO_2_/t)	Carbon efficiency	CSI
2018	10	10	9642a	97.0a	8.66a	22.81a
		30	9595a	104.4a	8.62a	22.70a
	**Average**		**9619D**	**100.7B**	**8.64D**	**22.75D**
						
	20	10	10044a	86.1b	9.02a	23.81a
		30	10117a	92.7a	9.09a	23.99a
	**Average**		**10080C**	**89.4C**	**9.05C**	**23.90C**
						
	30	10	11065a	74.8b	9.94a	26.33a
		30	10553a	85.3a	9.48a	25.06a
	**Average**		**10809B**	**80.0D**	**9.71B**	**25.69B**
	Local ratoon rice production model^&^	**11992A**	**111.54A**	**10.77A**	**28.62A**	
ANOVA	Cutting time (T)	374**	4.3**	0.34**	0.92**	
	Cutting height (H)	ns	3.5**	ns	ns	
	T*H	ns	ns	ns	ns	
2019	10	10	10552a	89.1b	9.48a	25.06a
		30	9922b	103.6a	8.91b	23.50b
	**Average**		**10237B**	**96.4B**	**9.19A**	**24.28A**
	20	10	10708a	78.1b	9.62a	25.45a
		30	9703b	91.2a	8.71b	22.96b
	**Average**		**10206B**	**84.7C**	**9.17A**	**24.20A**
	30	10	10629a	71.4b	9.55a	25.25a
		30	9910a	83.0a	8.90a	23.47a
	**Average**		**10270B**	**77.2D**	**9.22A**	**24.36A**
	Local ratoon rice production model	**11340A**	**117.06A**	**10.18A**	**27.01A**	
ANOVA	Cutting time (T)	1040**	4.4**	ns	ns	
	Cutting height (H)	317**	3.6**	0.28**	0.78**	
	T*H	ns	ns	ns	ns	

CF_S_, Carbon footprints; CPP, Crop primary productivity; CSI, Carbon sustainability index.

^&^Cutting time and heights of the local ratoon rice production model was 30 cm stubble height at the mature stage of the main crop.

Different small letter between means were assessed with the least significant difference (LSD0.05) at p ≤ 0.05.

* means significant at the 0.05 level, ** means significant at the 0.01 level, and ns means the correlation is not significant.

Carbon efficiency and carbon sustainability index was increase with cutting time delay, and there was significant difference among the treatment of cutting time in 2018 (p < 0.05). The carbon efficiency and carbon sustainability index of the FFRR was 9.94 and 26.33, respectively in 2018, and 9.55 and 25.25, respectively in 2019 with the treatment of 30 days after the flowering stage while the stubble height was 10 cm (Table 5).

## 5 Economic benefits of the FFRR

The net income (NI) of the FFRR was 30,577 CNY hm^−2^ in 2018 and 21,367 CNY hm^−2^ in 2019 in the treatment of 30 days after the flowering stage while the stubble height was 10 cm, and there were significant differences among the treatments of T and H (Table 6). A high NI depended mainly on a high rice grain yield of the ratoon crop. The grass income of the FFRR was 45,816 CNY hm^−2^ in 2018 and 36,605 CNY hm^−2^ in 2019 in the treatment of 30 days after the flowering stage and a stubble height of 10 cm, and there was significant difference among the treatment of cutting time in 2019 (*p* < 0.05). Similarly, a high cost–benefit ratio and labor productivity were realized in the treatment of 30 days after the flowering stage and a stubble height of 10 cm in 2018. Economic benefits of the local ratoon rice production model were lower (*p* < 0.01) than that of the treatment of 30 days after the flowering stage while the stubble height was 10 cm.

**Table 6:** Economic benefits and its index among different cutting time and cutting height in the forage–food dual-purpose ratoon rice cropping.

Year	Cutting time (days after FL)	Cutting height (cm)	Gross income (CNY hm^−2^)			O/I	NI (CNY hm^−2^)	CBR	LP (CNY person^−1^.hr^−1^)
			Ratoon crop	Main crop^$^	Total				
2018	10	10	17159b	23287a	40445a	2.65a	25207a	1.65a	52.52a
		30	19898a	17479b	37377b	2.45b	22139b	1.45b	46.12b
	**Average**		**18528A**	**20383C**	**38911B**	**2.55B**	**23673B**	**1.55B**	**49.32B**
	20	10	14266b	30319a	44586a	2.93a	29347a	1.93a	61.14a
		30	18156a	19201b	37357b	2.45b	22119b	1.45b	46.08b
	**Average**		**16211B**	**24760B**	**40971A**	**2.69A**	**25733A**	**1.69A**	**53.61A**
	30	10	13278b	32537a	45816a	3.01a	30577a	2.01a	63.70a
		30	16853a	19435b	36288b	2.38b	21050b	1.38b	43.85b
	**Average**		**15066C**	**25986A**	**41052A**	**2.69A**	**25813A**	**1.69A**	**53.78A**
	Local ratoon rice production model^&^		**16853D**	**19235D**	**36088C**	**2.37C**	**20850C**	**1.37C**	**43.44C**
ANOVA	Cutting time (T)		413**	863**	1074**	0.07**	1074**	0.07**	2.24**
	Cutting height (H)		417**	912**	1182**	0.08**	1181**	0.08**	2.46**
	T*H		ns	1580**	2046**	0.14**	2046**	0.14**	4.26**
2019	10	10	19049b	23515a	42564a	2.79a	27326a	1.79a	56.93a
		30	21597a	15353b	36951b	2.42b	21712b	1.42b	45.23b
	**Average**		**20323A**	**19434B**	**39758A**	**2.61A**	**24519A**	**1.61A**	**51.08A**
	20	10	13473b	26429a	39902a	2.62a	24664a	1.62a	51.39a
		30	14845a	18179b	33025b	2.17b	17786b	1.17b	37.06b
	**Average**		**14159B**	**22304A**	**36463B**	**2.39B**	**21225B**	**1.39B**	**44.22B**
	30	10	7876b	28729a	36605a	2.40a	21367a	1.40a	44.51a
		30	11519a	17758b	29277b	1.92b	14039b	0.92b	29.25b
	**Average**		**9698C**	**23244A**	**32941C**	**2.16C**	**17703C**	**1.16C**	**36.88C**
	Local ratoon rice production model		**11519C**	**22120A**	**33639C**	**2.21C**	**18401C**	**1.21C**	**38.33C**
ANOVA	Cutting time (T)		462**	1205**	1127**	0.07**	1127**	0.07**	2.35**
	Cutting height (H)		490**	750**	1002**	0.07**	1002**	0.07**	2.09**
	T*H		849*	1299*	ns	ns	ns	ns	ns

NI, net income; LP, Labo(u)r productivity; CBR, Cost-benefit ratio.

^&^Cutting time and heights of the local ratoon rice production model was 30 cm stubble height at the mature stage of the main crop.

^$^ Price of rice straw per t was 700 CNY.

Different small letter between means were assessed with the least significant difference (LSD0.05) at p ≤ 0.05.

* means significant at the 0.05 level, ** means significant at the 0.01 level, and ns means the correlation is not significant.

## 6 Discussion

### 6.1 Improving system productivity of the FFRR in practice

In this study, we performed an on-farm assessment of system productivity among different cutting times and stubble heights of rice. The findings of this study show that the straw yield of the ratoon crop increased with a cutting time delay and stubble height increase. The average maximum straw yield was 18.1 t hm^−2^ in the treatment of 30 days after the flowering stage and a stubble height of 10 cm (Table 4). Similarly, Ref. [5] reported that Zhunliangyou 608 could be used as ratoon rice in subtropical and temperate rice planting areas to produce good-quality forage by using a 30 cm stubble height for the first season when the grain had reached 80% maturity. However, forage yield rather than forage quality is more important for livestock farming in southern China, especially considering the supply shortage of fresh forage during winter; therefore, a lower stubble height is beneficial because of the higher forage yield. Our data in the forage quality experiment reported by Ref. [12] was indicated that whole plant rice silage could be used to partially replace whole plant corn silage in dairy cows diet, which could improve the immune capacity and reduce feeding costs of dairy cows and provide a good foundation for obtaining higher economic benefits. Compared with the local ratoon rice production model, the FFRR was produce forage and grain simultaneously for supporting the development of livestock farming and maintaining the supply of the food.

Cutting time and stubble height are two important factors affecting the rice yield and quality of ratoon crops. Reference [6] reported significant differences in ratoon crop grain yield between different cutting times and cutting heights and found a maximum ratoon crop grain yield at a stubble height of 40 cm in America. [11], working in Guangdong Province, southern China, reported that a 5 cm stubble height prolonged the ratoon crop growth duration and increased the spikelet number per panicle. In Hunan Province, central China, the cutting time and stubble height in the ratoon rice production system are about 30 days after the flowing stage and 30 cm, respectively. Our results indicate that the grain yield of the ratoon crop decreased with a cutting time delay and stubble height decrease (Table 4). The highest grain yield of the ratoon crop occurred with a cutting time of 10 days after the flowering stage and a cutting height of 30 cm. Therefore, the most suitable cutting time and cutting height depend on local climate conditions.

In FFRRs systems, improving the agricultural practices of optimal cutting time and cutting height can help balance the rice straw yield of the main crop for forage and the rice grain yield of the ratoon crop for unprocessed grain. The results of this study help to fill knowledge gaps about the performance of FFRR systems in China. Consequently, farmers can select the optimal strategy to satisfy the demands for unprocessed grain, forage, or both.

### 6.2 Improved practices improve the energy and carbon budgeting of FFRR systems

For cleaner production technology, reducing the carbon footprint, improving energy consumption and gas emissions, and maintaining soil health simultaneously are the major targets for fulfilling the sustainable production goals of agriculture [9]. Ratoon rice exhibits a significantly higher net energy ratio and benefit-to-cost ratio and a substantially lower yield-scaled global warming potential than the other two cropping systems (i.e., double-season and middle-season rice [4]). In this study, based on the same energy inputs, compared with the local ratoon rice production model, equivalent carbon emissions for the agricultural inputs used, as well as the inputs based on money, the high system productivity (including straw yield and ratoon rice yield) achieved by improving cutting time and cutting height led to high net energy and EUE. However, there was no alteration of CPP, possibly because the CPP of each rice cultivar was relatively constant under the same ecological conditions and cultivation. Thus, there was no effect by cutting time or cutting height.

### 6.3 Advantages of FFRR systems

In FFRR systems, the rice main crop (including the rice stem and grain) is harvested to help satisfy the forage need for large domestic animals and the rice ratoon crop is harvested to help satisfy the demand for high-quality unprocessed grain to feed people. The ability to increase the forage yield by improving the cutting time and cutting height when there is enough high-quality unprocessed grain or decrease the forage yield when unprocessed grain is insufficient is a major advantage of the system, compared with the local ratoon rice production model. Thus, FFRR is an effective way to ensure both forage security and unprocessed grain security, especially in relatively impoverished regions of Southeast Asia, Africa, and South America.

## 7 Conclusions

Compared with the local ratoon rice production model, FFRR was shown high total output energy, net energy, energy use efficiency, energy productivity and energy profitability. There was significant difference (p < 0.05) between the treatment of 30 days after the flowering stage while the stubble height was 10 cm and the local ratoon rice production model. Our data was shown that economic benefits of the local ratoon rice production model were lower (*p* < 0.01) than that of the treatment of 30 days after the flowering stage while the stubble height was 10 cm. Therefore, FFRR systems can be optimized effectively by selecting the ideal cutting time and cutting height, from the perspectives of carbon footprint and economic benefits, harvesting at 30 days after the flowering stage and with a stubble height of 10 cm was the optimal cultivation practice of the FFRR.

In this study, only one high-yielding cultivar were studied according to local high-yielding cultivation technology, there was significant difference of rice aboveground biomass and grain yield among the cultivars, moreover, fertilizer (for example: nitrogen rates and its application) management was affected significantly rice aboveground biomass and grain yield, further research was needs to testify the conclusions from more rice genotype or cultivars (high-yielding or high-quality) and nitrogen management.

## CRediT author statement

Chen Yuanwei, Zheng Huabin, Wang Weiqin, Tang Qiyuan: Conceived and designed the experiments; Performed the experiments; Analyzed and interpreted the data; Wrote the paper.

Zheng Huabin, Wang Weiqin: Contributed reagents, materials, analysis tools or data.

## Funding

The study was financially supported by the Earmarked Fund for China Agriculture Research System (No. CARS-01-27). Funds from the Ministry of Agriculture & Rural affairs.

## Data availability statement

The datasets generated during and/or analyzed during the current study are available from the corresponding author on reasonable request.

## Institutional review board statement

Not applicable.

## Informed consent statement

Not applicable.

## Declaration of competing interest

The authors declare that they have no known competing financial interests or personal relationships that could have appeared to influence the work reported in this paper.
